# To the Editors of the Medical and Physical Journal

**Published:** 1802-03

**Authors:** Thomas Docker

**Affiliations:** Dover


### *To the Editors of the Medical and Phyfical Journal.*

GENTLEMEN,

ACCIDENT prevented my feeing the 34th Number of the Medical and Phyfical Journal till within thefe few days, or I fhould certainly have anfwered the queries of Mr. Ward, refpecting the cafe of John Bayley, inferted in your Journal for February, 1800, in which I had fuccefsfully ufed the opiate friction.

I am happy that Mr. Ward's queftions gives me now an opportunity of correcting an error of the prefs with refpect to the quantity of camphire and oil which was ufed in each portion of the liniment in the cafe of Bayley. The following was the quantity ufed at each friction, viz. camphire half a drachm, ol. oliv. two drachms, the tincture of opium *only* was increafed or diminifhed as circumftances indicated.

In anfwer to the other queftions of Mr. Ward, whether any fyphilitic fymptoms appeared fince the bubo was healed? and whether mercury had been employed? I can only inform him, that during the period he continued in the hofpital after his recovery, no venereal fymptoms whatever appeared; but he had taken mercury to a confiderable extent previous to his admiffion into the hofpital, but as it was exhibited during the time that he was in Holland, and partly on board a tranfport, where it was impoffible proper attention could be paid to him, I attributed the bad ftate of his health and the fphacelating appearance of the bubo principally to that circumftance.

From being much emaciated he became in a very fhort time after his recovery extremely corpulent, and left the hofpital to join his regiment in the beft poffible ftate of health. I have fince that heard no more account of him.

The opiate friction was ufed in a few fimilar cafes with that of Bayley, but not with the fame *ftriking* advantages, yet was of evident fervice in arrefting the fpreading of gangrene, and allaying irritation. Lately I have had no opportunity of trying its efficacy further, but am convinced of its good and powerful effects.

I fincerely wifh Mr. Ward every fuccefs in his indefatigable endeavours to afcertain the modus operandi of opium when introduced by friction, and he may depend upon my communicating the refult of any future trials I may have an opportunity of making with it.

I am, &c.

